# Ultrasensitive Electrochemical Detection of *Salmonella typhimurium* in Food Matrices Using Surface-Modified Bacterial Cellulose with Immobilized Phage Particles

**DOI:** 10.3390/bios14100500

**Published:** 2024-10-14

**Authors:** Wajid Hussain, Huan Wang, Xiaohan Yang, Muhammad Wajid Ullah, Jawad Hussain, Najeeb Ullah, Mazhar Ul-Islam, Mohamed F. Awad, Shenqi Wang

**Affiliations:** 1 Advanced Biomaterials and Tissue Engineering Center, College of Life Science and Technology, Huazhong University of Science and Technology, Wuhan 430074, China; wajidusafzai@hust.edu.cn (W.H.); m202071916@hust.edu.cn (H.W.); d202180745@hust.edu.cn (X.Y.); 2 Department of Pulp & Paper Engineering, College of Light Industry and Food Engineering, Nanjing Forestry University, Nanjing 210037, China; 3 Department of Biomedical Engineering, College of Life Science and Technology, Huazhong University of Science and Technology, Wuhan 430074, China; jawadusafzai5@gmail.com; 4 Department of Chemical and Biomolecular Engineering, University of Tennessee, Knoxville, TN 37996, USA; lzn122@utk.edu; 5 Department of Chemical Engineering, Dhofar University, Salalah 211, Oman; mulislam@du.edu.om; 6 Department of Biology, College of Science, Taif University, Taif 21944, Saudi Arabia; m.fadl@tu.edu.sa

**Keywords:** bacterial cellulose, *S. typhimurium*, bacteriophage-based detection, electrochemical biosensor, selectivity, sensitivity, stability

## Abstract

The rapid and sensitive detection of *Salmonella typhimurium* in food matrices is crucial for ensuring food safety. This study presents the development of an ultrasensitive electrochemical biosensor using surface-modified bacterial cellulose (BC) integrated with polypyrrole (Ppy) and reduced graphene oxide (RGO), further functionalized with immobilized *S. typhimurium*-specific phage particles. The BC substrate, with its ultra-fibrous and porous structure, was modified through in situ oxidative polymerization of Ppy and RGO, resulting in a highly conductive and flexible biointerface. The immobilization of phages onto this composite was facilitated by electrostatic interactions between the polycationic Ppy and the negatively charged phage capsid heads, optimizing phage orientation and enhancing bacterial capture efficiency. Morphological and chemical characterization confirmed the successful fabrication and phage immobilization. The biosensor demonstrated a detection limit of 1 CFU/mL for *S. typhimurium* in phosphate-buffered saline (PBS), with a linear detection range spanning 10^0^ to 10^7^ CFU/mL. In real samples, the sensor achieved detection limits of 5 CFU/mL in milk and 3 CFU/mL in chicken, with a linear detection range spanning 10^0^ to 10^6^ CFU/mL, maintaining high accuracy and reproducibility. The biosensor also effectively discriminated between live and dead bacterial cells, demonstrating its potential in real-world food safety applications. The biosensor performed excellently over a wide pH range (4–10) and remained stable for up to six weeks. Overall, the developed BC/Ppy/RGO–phage biosensor offers a promising tool for the rapid, sensitive, and selective detection of *S. typhimurium*, with robust performance across different food matrices.

## 1. Introduction

Pathogenic microorganisms produce toxins or secondary metabolites that cause foodborne diseases. Among these pathogens, *Salmonella* [1] is particularly notable for its extremely low infectious dose, requiring only one colony-forming unit (CFU) to cause illness. *Salmonella* is a major contributor to diarrheal diseases worldwide [2], with common symptoms including diarrhea, abdominal pain, fever, and cramps, typically developing within 12–72 h after infection [3]. The disease is commonly associated with the consumption of various food matrices, including those of animal origin (poultry, meat, dairy products, and seafood products) and non-animal origin (fresh vegetables, fruits, and cereals) [4]. Therefore, developing a practical method for the early and selective detection of *S. typhi* in food samples is crucial for ensuring food safety and reducing the risk of human exposure to this bacterial threat [5].

Conventional detection methods, such as culture techniques and microscopic examination, have several limitations. For instance, techniques such as enzyme-linked immunosorbent assay (ELISA) and polymerase chain reaction (PCR) require trained personnel and costly reagents, making them unsuitable for on-site detection [6]. To address this, biosensors with specific bio-recognition elements have emerged as a key solution for the on-site detection of *S. typhimurium* [7]. In recent years, significant efforts have been made to develop various types of biosensing platforms, including optical, piezoelectric, and electrochemical, for the rapid and specific detection of pathogens using nucleic acids, antibodies, enzymes, and other bioprobes [8,9,10]. Among novel sensing platforms, phage-based electrochemical detection has gained widespread acceptance owing to its high accuracy, specificity, and short detection time [11]. Additionally, phages are readily available, highly tolerant to extreme pH, temperature, and organic solvents, and are more stable than other bioprobes [12].

Immobilizing bacteriophages on an appropriate substrate is crucial to ensure that the phage tail retains its ability to infect the host bacterium when used as a bioprobe [8]. To optimize the interaction between phage tail spikes and bacterial cell wall receptor-binding proteins (RBPs), a phage is oriented with its tail pointing up and its head pointing down [13]. This orientation can be achieved through various methods including genetic modification [14], simple physical adsorption [15], and electrostatic interactions [16]. Among these, electrostatic interactions are a particularly effective and sustainable strategy for immobilization. In this approach, the negatively charged proteins of the phage capsid form a stable chemical bond with a positively charged substrate, leaving the tail exposed to capture bacteria [14,17]. To date, various materials such as carbon-based nanomaterials [18], metal oxides [19], and polymers [20] have been used to anchor phages in the development of electrochemical biosensors. Bacterial cellulose (BC), produced by the genus *Komagataeibacter* and others [21], as well as cell-free enzyme systems, was chosen as the substrate for immobilizing phage particles. BC offers exceptional properties, including a highly porous and fibrous structure, high humidity, biocompatibility, flexibility, and strength [22]. These characteristics have made BC a valuable material for various applications, such as tissue engineering, bone regeneration, drug delivery, enzyme immobilization, and sensor development, in both nanomaterial and polymer forms [15,23,24]. However, pristine BC lacks the necessary conductivity and carries a net negative charge owing to the presence of -OH groups [25]. Electrical conductivity can be imparted to BC by introducing conductive materials such as polypyrrole (Ppy) and reduced graphene oxide (RGO) into its matrix. Ppy is an important conjugated polymer known for its inherent conductivity, adjustable surface properties, exceptional biocompatibility, and ease of polymerization at low potentials [26,27]. It can be employed as a versatile support matrix for biomolecules such as DNA [28], enzymes [29], and antibodies [30] and has also been used as a surface for cell growth [31]. It can be synthesized using various solvents, including water or chemically with oxidants such as ammonium persulfate (APS) and iron chloride (FeCl_3_), along with dopants such as cetyltrimethylammonium bromide (CTAB), p-toluenesulfonic acid (TSA), and sodium dodecyl sulfate (SDS) [32,33,34]. The versatility of Ppy in electrochemical processes makes it a promising candidate for use in supercapacitors and battery electrodes [35] because of its electrical conductivity, ease of synthesis, low cost, and stability under various environmental conditions [36]. The -OH groups on the surface of BC can interact with pyrrole monomers through hydrogen bonding, facilitating the in situ polymerization of Ppy. The unique structure of BC makes it an ideal nanotemplate for preparing flexible freestanding electrodes using Ppy [37,38]. However, the poor mechanical properties of Ppy limit its applications as a flexible material. To address this, researchers have enhanced its mechanical and electrochemical characteristics by incorporating various nanomaterials such as gold nanoparticles [39] and carbon nanofibers [40] to form unique Ppy/nanocomposites. Adding a rational amount of RGO, which is known for its high electrical conductivity, large specific surface area, and excellent electrochemical stability, can further improve the performance of this freestanding film [41]. Additionally, the combination of the ultrafine network of BC, nitrogen in the Ppy ring, and high-conductivity RGO could enhance the surface area and overall performance of the composite material.

The study aimed to develop a novel, rapid, cost-effective, accurate, and efficient phage-based detection platform for *S. typhimurium* in food samples. The polymerizing of Ppy, combined with the fibrous structure of BC, along with the presence of an amine group on the pyrrole ring and positive charges along the Ppy backbone, could facilitate the attachment and immobilization of negatively charged phage capsid proteins for use as a biointerface [42,43,44,45]. The newly developed BC/Ppy/RGO–phage biointerface was characterized for structural and chemical topographies through FE-SEM, XRD, FTIR, XPS, and confocal microscopy. Additionally, the anti-*Salmonella* and lytic activities of the immobilized phages were analyzed. The electrochemical performance of the biointerface was evaluated using cyclic voltammetry (CV) and differential pulse voltammetry (DPV). When the immobilized phages on BC/Ppy/RGO specifically recognize and capture *S. typhimurium*, they induce phage-mediated cell lysis, releasing cellular components. This will result in an increase in electrochemical signals, facilitating the detection of bacterial cells. The developed biosensor was evaluated for specificity, selectivity, and the ability to discriminate between live and dead bacteria in a mixture of live/dead cells, as well as in the presence of both host and non-host bacterial cells. Additionally, the reproducibility, storage-based stability, and detection of *S. typhimurium* in real samples were investigated. The developed BC/Ppy/RGO–phage biosensor is an ideal platform for the early detection of *S. typhimurium* with high sensitivity, specificity, and accuracy in food matrices to prevent various foodborne illnesses.

## 2. Materials and Methods

### 2.1. Materials

Chemicals, including glucose, disodium hydrogen phosphate, citric acid, yeast extract, bacteriological peptone, sodium hydroxide, calcium carbonate, and a premix of Luria–Bertani (LB), were obtained from Alfa Aesar (Beijing, China). Graphene oxide was acquired from XFNANO Materials Technology (Shanghai, China). Pyrrole and p-toluenesulfonic acid (p-TSA) were purchased from the Shanghai Biochemical Technology Co., Ltd. (Shanghai, China). Ferric hexahydrate (FeCl_3_·6H_2_O) and sodium hydroxide were obtained from Shanghai Sinopharm Chemical Reagent Co., Ltd. (Shanghai, China). All chemical reagents were of analytical grade and were used without further purification, unless otherwise stated.

### 2.2. Bacterial Strains and Cell Culture

*Salmonella typhimurium* (CCTCC AB204062) was used as the phage host bacterial strain, whereas *Staphylococcus aureus* (CCTCC AB2013186), *Pseudomonas aeruginosa* (LC-B24), *Yersinia* pseudotuberculosis (LC-B7), and *Klebsiella* pneumoniae (ATCC 21196) were used as non-host bacterial strains. *Gluconacetobacter xylinus*, kindly provided by the 3-Bio Lab in the Department of Biomedical Engineering at Huazhong University of Science and Technology, was used for bacterial cellulose production.

The phage host strain, *S. typhimurium*, was streaked onto LB agar plates (3× LB) and incubated at 37 °C for 12 h to allow colony formation. A single colony was selected and incubated in LB broth (3× LB) at 37 °C for 24 h. The resulting broth culture was stored at 4 °C for subsequent use in bacteriophage isolation and purification. The non-host bacterial strains, *P. aeruginosa*, *S. aureus*, *Y. pseudotuberculosis*, and *K. pneumoniae*, were cultured in LB liquid media according to their respective protocols and stored at 4 °C for future use.

### 2.3. Isolation and Purification of S. typhimurium-Specific Phage

A specific lytic phage was isolated from sewage water collected from a pig dairy farm using a modified version of a previously reported protocol [46]. Briefly, a 400 mL sample of sewage water was taken from a pig farm in Henan Province, China, and left undisturbed to allow dense particles to settle. Solid debris and particles were removed by centrifuging the sample at 5000× *g* for 10 min at room temperature. The supernatant was then filtered through a 0.22 µm syringe-driven filter and stored at 4 °C. The filtered sample was used to isolate and amplify the phage on a double-layer agar plate. Purified phages were quantified in terms of plaque-forming units per mL (PFU/mL). The *S. typhimurium*-specific phages were further characterized by a plaque spot assay, transmission electron microscopy (TEM) to observe phage morphology (see Supplementary Materials).

### 2.4. Reduction of Graphene Oxide

The reduction of GO to RGO was performed using ascorbic acid, a safe and non-toxic reducing agent, following a previously established protocol [47,48]. Briefly, a mixture was prepared by combining equal dispersions of 40 mL GO (0.1 mg) and 40 mg ascorbic acid (0.1 M). The mixture was heated to 95 °C with continuous stirring for 2 h. After the reaction, the solution was washed several times with deionized water and ethanol, followed by centrifugation at 1000× *g* for 5 min. The supernatant was discarded, and the remaining RGO was oven-dried at 60 °C.

### 2.5. Bacterial Cellulose Production and Purification

BC membranes were produced following a previously reported method [49]. A *G. xylinus* strain, used with special permission from the 3-Bio Lab., was inoculated into sterilized Hestrin–Schramm (HS) growth medium containing the following (g/L): glucose (20.0), yeast extract (5.0), peptone (5.0), disodium hydrogen phosphate (2.5), and citric acid monohydrate (1.15). The culture was maintained at pH 6.5, 30 ± 1 °C, and agitated at 100 ± 2 rpm for 24 h in 500 mL Erlenmeyer flasks. Following this, 3–5% (*v*/*v*) of the culture was transferred to freshly prepared 1 L HS medium and incubated statically at 30 °C for 7–10 days. The resulting BC membranes were thoroughly washed with deionized water and then immersed in an ethanolic solution of NaOH (0.3 M) at 80 °C for 90 min to remove impurities. The membranes were rinsed with deionized water until the pH reached 7.0. Purified BC sheets were stored at 4 °C until further use.

### 2.6. Development of BC/Ppy/RGO Biointerface

The BC membrane was surface-modified with Ppy and RGO using a slightly modified version of a previously reported method [50,51]. The conductive BC/Ppy/RGO bio-interface was developed via in situ chemical oxidative polymerization of Py with RGO on the surface of BC. FeCl_3_·6H_2_O served as the oxidant, and p-toluenesulfonic acid monohydrate (p-TSA, C_7_H_8_O_3_·H_2_O) was used as a dopant. Briefly, the wet BC membrane was cut into strips (3 cm × 2 cm) and swollen in deionized water by boiling. The swollen BC hydrogel was then mechanically wet-starched at 30% at a rate of 1 mm/min. The BC was subjected to in situ chemical oxidative polymerization of Py for a fixed period in the presence of FeCl_3_·6H_2_O and C_7_H_8_O_3_·H_2_O (p-TSA) with various concentrations of RGO. For the polymerization process, Py (0.2 mL) and RGO (0.015 g/L) at different concentrations (0.1, 0.5, and 0.1%) were added to a 20 mL solution of p-TSA (0.2 g) in double-distilled water (ddH_2_O) and sonicated for 40 min to achieve a uniform dispersion. Meanwhile, 1 g of FeCl_3_·6H_2_O was dispersed in 5 mL of ddH_2_0, and the wet BC membrane was soaked in this solution to fully absorb the oxidant. The membrane was then immersed in Py, p-TSA, and RGO suspensions, shaken for 15 min, and subsequently transferred to 4 °C to initiate polymerization for 1 h. The same procedure was followed to prepare Ppy and BC/Ppy composites. After polymerization, the resulting BC/Ppy/RGO composite films were washed several times with deionized water to remove unreacted components. Finally, the films were freeze-dried for 16–18 h to obtain freestanding membranes. The BC/Ppy/RGO composites were labeled according to the RGO concentration as BC/Ppy/RGO-I, II, and III for 0.1%, 0.5%, and 1%, respectively. The composites were stored under dry conditions until further use.

### 2.7. Immobilization of S. typhimurium-Specific Phages on BC/Ppy/RGO Biointerface

*S. typhimurium*-specific phages were immobilized on the BC/Ppy/RGO-I, II, and III biointerfaces of 1 × 1 cm dimension. The biointerfaces were submerged in a phage solution in PBS with a titer of 3.7 × 10^9^ PFU/mL and incubated overnight at room temperature with gentle shaking. After incubation, the biointerfaces were carefully removed, gently rinsed, and washed with PBS to eliminate loosely attached or unbound phages. The resulting phage-immobilized biointerfaces were labeled BC/Ppy/RGO (I)–phage, BC/Ppy/RGO (II)–phage, and BC/Ppy/RGO (III)–phage. Finally, the biointerfaces were freeze-dried, sealed, and stored at 4 °C until further use.

### 2.8. Characterization of BC/Ppy/RGO Biointerface

The morphology of pristine BC and its various composites modified with Ppy and RGO, both before and after immobilization of *S. typhimurium*-specific phages, was observed using a field-emission scanning electron microscope (FE-SEM) (Nova Nano FE-SEM 450, FEI Company, Hillsboro, OR, USA). Small pieces of pristine BC, Ppy, BC/Ppy, BC/Ppy/RGO, and BC/Ppy/RGO–phage films were adhered to a carbon-tape-coated stub using double-sided carbon tape. The samples were then sputter-coated with gold for 120 s by using a sputtering unit (Bal-Tec SCD005 Sputter Coater, Shanghai, China). Micrographs were captured, and FE-SEM was used to acquire the elemental mapping images of the sample. The fiber diameters in the micrographs were determined using the ImageJ software (ImageJ 1.52n, NIH-USA), with averages calculated from measurements at different locations. The crystal structures of the biointerfaces were characterized using X-ray diffraction (XRD) (PANalytical B.V., Almelo, The Netherlands). The physicochemical properties and functional group variations in all the samples were characterized using Fourier-transform infrared (FTIR) spectroscopy (VERTEX 70, Bruker Corporation, Ettlingen, Germany). Additionally, X-ray photoelectron spectroscopy (XPS) was used to analyze the chemical composition of the samples.

### 2.9. Plaque Assay and Cell Lysis Efficiency of Immobilized Phage

For the plaque assay, 100 µL of an overnight *S. typhimurium* culture was mixed with 4 mL of top agar and spread onto LB bottom agar plates. After the agar solidified, the pristine BC, various BC/RGO composite films, and BC/RGO–phage films were carefully placed in the center of the culture plates. Pristine BC, BC/ppy, and BC/Ppy/RGO were used as controls. All plates were incubated overnight at 37 °C, and plaque formation was observed around the edges of the composite films.

To assess the cell lysis efficiency, the above-mentioned samples were inoculated into a pre-incubated *S. typhimurium* (OD_600_) culture and shaken at 150 rpm at 37 °C. A pure *S. typhimurium* culture served as a negative control, while an *S. typhimurium* culture inoculated with phage was used as the positive control. Readings were taken every hour for 11 h, and OD_600_ was determined every hour for a total of 11 h of infection. Finally, the growth curve was analyzed to determine the cell lysis efficiency of the BC/Ppy/RGO–phage biointerface.

### 2.10. Fluorescent Characterization of Immobilized Phages

The density of immobilized phage particles on the developed BC/Ppy/RGO biointerface was determined by fluorescence imaging following standard protocols [52,53]. Briefly, immobilized phages on different composite films (1 × 1 cm) were stained with 10 µL of diluted fluorescent nucleic acid stain (15 × SYBER Gold, Life Technologies, Carlsbad, CA, USA) solution for 25 min at room temperature in the dark. The excess stain was washed off, and the films were placed on a labeled glass slide. Before adding a cover slip, a drop of antifading agent was applied. The samples were then observed under a confocal microscope (Olympus FV1000, OLYMPUS Co., Ltd., Beijing, China) with a 60× oil immersion lens. The surface densities of the phage particles immobilized on the films were measured, and the density (phages/μm^2^) was calculated for each image. This process was repeated thrice to ensure accuracy.

### 2.11. Electrochemical Characterization

The BC/Ppy/RGO–phage biointerface was cut into 0.5 cm × 1 cm pieces and analyzed by cyclic voltammetry (CV) and differential pulse voltammetry (DPV) to evaluate its electrochemical performance. Electrochemical characterization was conducted using a conventional electrode setup consisting of a Pt wire as the auxiliary electrode, Ag/AgCl reference electrode, and BC/Ppy/RGO–phage as the working electrode. All measurements were performed at 25 ± 2 °C in a voltammetry cell containing 10 mL of an electrocatalytic solution composed of 5 mM 1:1 K_3_[Fe(CN)_6_] and K_4_[Fe(CN)_6_] with 0.1 M KCl. To optimize the electrode, the response time of the biosensor was determined by measuring the current response every 5 min during a 10–60 min incubation period. The BC/Ppy/RGO–phage electrode was placed in 10 mL of the electrocatalytic solution, and a specific dilution of S. typhimurium was added. The DPV-based current response was measured at 5 min intervals. For the sensitivity testing, the electrode was placed in a BPS solution at different pH levels (3–10), each containing the same dilution of *S. typhimurium*. The electrode was incubated, and the current was recorded to assess the pH-dependent variations in the current response. The optimized biosensor was used to detect *S. typhimurium* in BPS at various concentrations.

### 2.12. Specificity, Reproducibility, and Stability of BC/Ppy/RGO Biosensor

To evaluate the specificity and selectivity of the developed biosensor for electrochemical detection of *S. typhimurium* (4.2 × 10^6^ CFU/mL), tests were conducted against non-specific bacterial strains, including *P. aeruginosa* (3.07 × 10^6^ CFU/mL), *Y. pseudotuberculosis* (3.2 × 10^6^ CFU/mL), *K. pneumoniae* (2.05 × 10^6^ CFU/mL), and *S. aureus* (1.76 × 10^6^ CFU/mL). These bacteria were grown in their respective media and centrifuged, the supernatant was discarded, and the pellet was resuspended in separate PBS solutions and a mixed bacterial solution to record the biosensor response. The reproducibility of the BC/Ppy/RGO–phage biosensor was determined under identical conditions, and five different BC/Ppy/RGO–phage biointerfaces were employed to detect *S. typhimurium* (4.2 × 10^6^ CFU/mL). Both fresh and used biosensors were stored at 4 °C to assess the stability and effectiveness of the biosensor. The capture and sensing responses of S. *typhimurium* were recorded on DPV for up to five weeks.

### 2.13. Biosensor Application in Real Sample and Live/Dead Cell Discrimination

To assess the accuracy of the BC/Ppy/RGO–phage biosensor in milk and chicken as representative food matrices, we followed established protocols with slight modifications [8]. Briefly, sterilized milk was obtained from a local market, and 25 mL of the milk sample was heated to 60 °C for 25 min. After heating, the samples were centrifuged at 10,000× *g* for 15 min to remove the upper and lower fat layers. The middle layer was then subjected to additional centrifugation, and the supernatant was filtered through a 0.45 µm filter followed by a 0.22 µm filter for sterilization. Subsequently, the sample was diluted to 1:20 (*v*/*v*) in PBS and processed for *S. typhimurium* detection at various concentrations. Similarly, chicken samples were cut into (1 × 1 cm^2^) pieces in sterilized pottery dishes and exposed to UV light for 30 min to ensure complete surface sterilization [9]. The chicken pieces were artificially contaminated with *S. typhimurium* (200 µL) and allowed to dry in a biosafety cabinet for 30 min. The samples were divided into two groups: one group underwent culture plating for bacterial enumeration, and the other was tested using the biosensor for detection.

To distinguish between live and dead bacteria, DPV responses were recorded for *S. typhimurium* samples that had been treated at 100 °C for 25 min (dead cells), freshly grown bacteria (live cells), and a mixture of both (live/dead cells). To assess the recovery of the developed biosensor, *S. typhimurium* was inoculated into the samples, and the bacterial density was analyzed using both the plate count method and the developed phage-based biosensor.

## 3. Results and Discussion

### 3.1. Principle and Design of the Phage-Based Biosensor

The design of current biosensors involves several key steps: production of BC, fabrication of a BC/Ppy/RGO composite, isolation and purification of *S. typhimurium*-specific phage particles, and immobilization of these phages on the biointerface for use as a phage-based biosensor to electrochemically detect *S. typhimurium* in various food matrices.

**Figure 1:**
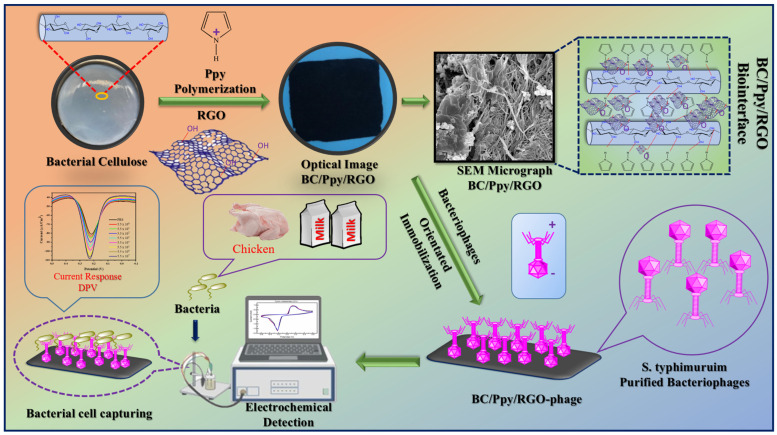
Fabrication of BC/Ppy/RGO composite. Immobilization of the *S. typhimurium*-specific phages to develop a BC/Ppy/RGO-phage biointerface for the electrochemical detection of *S. typhi* in milk and chicken samples using DPV.

The prepared BC substrate was first allowed to swell in water, after which the absorbed water was removed by mechanical pressing. The BC hydrogel was then immersed in an Fe^3+^ solution, which acted as an oxidant, facilitating the coating of the BC substrate. A mixture of Ppy, RGO, and aqueous p-TSA was then added to the BC matrix, leading to the polymerization of Ppy and RGO on the BC surface. After drying, the pristine BC, BC/Ppy, and BC/PPY/RGO composites were obtained. The detailed procedure and optimized protocol are shown in Figure S1. The *S. typhimurium*-specific phage was isolated and purified from dairy farm sewage water using the double-layer agar method (Figure S2), and its lytic activity against the host bacterial strain was confirmed (Figure S3). TEM revealed that the phages were tailed, with a hexagonal capsid approximately 86 nm in size and a tail length of 112 nm (Figure S4). The phage showed high specificity and lytic activity towards *S. typhimurium* (Table S1). Previous studies have shown that the design of paper-based electrodes with high mechanical and electrochemical performances is challenging. However, selecting an appropriate substrate and optimizing the structural design can simultaneously achieve these properties. In this study, a purified BC sheet was used as the substrate and modified with Ppy (Figure S5) and RGO via in situ oxidative polymerization. The ultra-fibrous and porous structure of BC promotes Ppy polymerization and RGO impregnation, thereby imparting conductivity, as previously reported [50,54]. The polycationic nature of Ppy also provided an attractive force for the negatively charged phage heads, enabling successful phage immobilization on the BC-based biointerface through electrostatic interactions between the phage capsid heads and Ppy-modified BC fibers [55,56,57]. Polymerized Ppy on the BC surface not only facilitates phage attachment but also enhances biosensor performance owing to its excellent properties when combined with RGO, such as electrical conductivity, surface flexibility, and biocompatibility [27,58]. The -OH groups on the BC surface form hydrogen bonds with Ppy, and RGO enhances the adhesion between BC and Ppy/RGO, resulting in a stable composite [59]. Additionally, both RGO and Ppy have a delocalized π-electron system that allows π-π stacking interactions with BC, thereby improving the conductivity and mechanical properties [60,61,62]. The mixing of RGO with Ppy during the in situ oxidative polymerization process in the presence of BC forms a uniform, lightweight, and conductive flexible membrane that can bend at large angles (Figure S6A,B). This suggests that Ppy polymerization combined with RGO on the BC biointerface could facilitate the immobilization of oriented phages, interacting with the negatively charged phage capsid while exposing the positively charged tail RBPs to capture bacteria. The highly porous structure of BC, cationic Ppy, and residual -COOH groups on RGO maximized phage particle immobilization. When the immobilized phages on BC/Ppy/RGO specifically captured and adsorbed *S. typhimurium*, they induced phage-mediated cell lysis and the release of cellular components, leading to an increase in electrochemical signals, which enabled bacterial cell detection. This process forms the basis for phage-based biosensors. The fabrication of BC/Ppy/RGO, isolation and purification of specific phages, and phage-based electrochemical detection of *S. typhimurium* are illustrated in Figure 1.

### 3.2. Morphology and Chemical Structure of the BC/Ppy/RGO–Phage Biointerface

**Figure 2:**
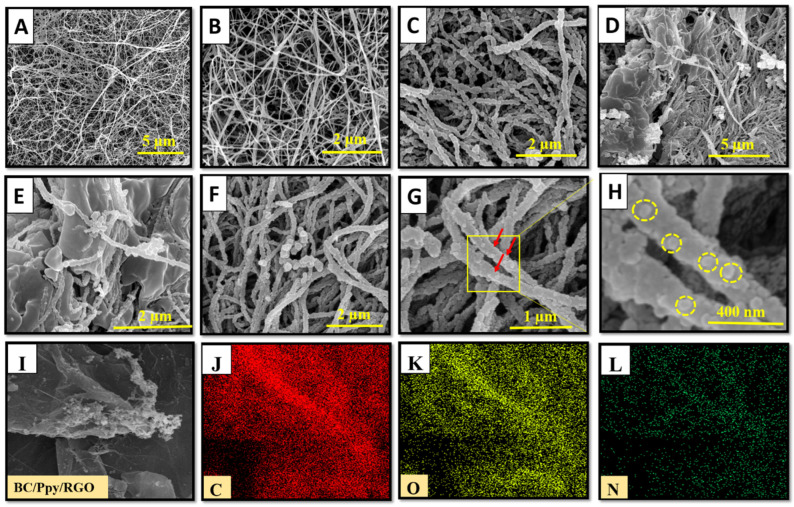
FE-SEM investigation of pristine BC before and after modification with Ppy, RGO, and immobilized phages. (**A**,**B**) Pristine BC at different magnifications, (**C**) Ppy polymerization on BC, (**D**,**E**) BC/Ppy/RGO at different magnifications, and (**F**,**G**) show the immobalized phages on BC/Ppy/RGO biointerface and red color arrows represent the individual phage particles. (**H**) Magnified image of the phage particles attached to the BC/Ppy/RGO shown in rectangular and the yellow circles represent individual phage particle. Elemental mapping images of BC/Ppy/RGO (**I**), carbon (**J**), oxygen (**K**), and nitrogen (**L**).

The structural morphologies of the pristine BC, BC/Ppy, BC/Ppy/RGO, and BC/Ppy/RGO–phage biointerfaces were observed using FE-SEM (Figure 2A–H). The FE-SEM micrographs revealed that pristine BC exhibited a 3D nano-network structure composed of loosely arranged fibrils with a porous, web-like architecture. This type of morphology has been observed in previous studies of pristine BC [63,64]. The pore size in the pristine BC ranged from a few micrometers to >1 µm, with submicron pores distributed throughout the 3D network (Figure 2A,B). These pores allow electrolytes to penetrate the entire web-like structure of BC, providing sufficient space for the uniform loading of conductive materials such as Ppy and RGO into the BC matrix, resulting in a compact, conductive structure. Similarly, as shown in Figure 2C, the BC/Ppy composite film maintained a porous web-like structure. Ppy particles, which formed small aggregates through in situ oxidative polymerization, were evenly distributed within the 3D fibril architecture of the BC. The hydrogen bond between the -OH groups of BC and Ppy acted as a traction force, promoting sufficient Ppy growth on the BC surface. This uniform deposition ensured no agglomeration or poor adhesion of Ppy nanoparticles on the BC membrane. In the case of the BC/Ppy/RGO composite (Figure 2D), RGO was clearly integrated with the BC/Ppy space within the fibril structure. The excellent contact between RGO and the Ppy nanoparticles on the BC surface is crucial for enhancing the conductivity of the biointerface. Additionally, the cross-linked porous networks in the BC/Ppy/RGO composite provided sufficient space for phage immobilization and attachment. The integration of RGO into the conductive BC/Ppy matrix facilitated rapid electron transfer and further improved conductivity. The polymerization of polycationic Ppy on the BC surface also played a significant role in orienting the negatively charged phage heads towards the positively charged biointerface. This orientation was optimal for immobilization, ensuring that the tail fibers were exposed and were capable of capturing bacterial cells. When the phage heads approached the interface surface, strong and stable immobilization was achieved through electrostatic interactions between Ppy and the phage head as well as the formation of amide bonds between the phage head proteins and -COOH groups. Figure 2F–H illustrate the immobilization of phages on the BC/Ppy/RGO surface at different magnifications, with yellow circles highlighting the immobilized phage particles. Additionally, the elemental compositional analysis of the BC/Ppy/RGO composite (Figure 2I–L) confirmed that C, O, and N were uniformly distributed, indicating the successful fabrication of the BC/Ppy/RGO composite.

**Figure 3:**
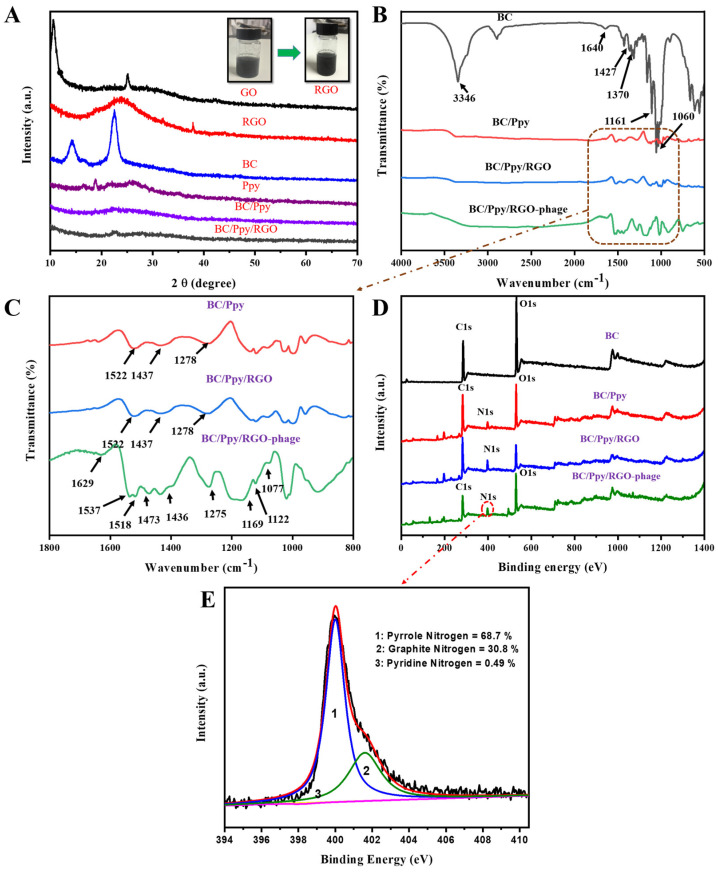
Characterization of modified BC composites and BC-phage biointerfaces. (**A**) XRD patterns of GO, RGO, BC, Ppy, BC/Ppy, and BC/Ppy/RGO and physiological changes of GO in RGO; (**B**) FT-IR spectra of BC, BC/Ppy, BC/Ppy/RGO, and BC/Ppy/RGO-phage; (**C**) Selected magnified area in (**B**) FT-IR-fingerprint of BC/Ppy, BC/Ppy/RGO, and BC/Ppy/RGO-phage ranging from 1800 to 800 cm^−1^. (**D**) XPS wide-scan patterns of BC, BC/Ppy, BC/Ppy/RGO, and BC/Ppy/RGO–phage. (**E**) N 1s core-level spectra of BC/Ppy/RGO-phage biointerface.

The reduction of GO to RGO, along with the crystallinity of the fabricated BC, Ppy, BC/Ppy, and BC/Ppy/RGO composites, was analyzed using XRD. As shown in Figure 3A, the XRD peak of GO at 2θ = 11.1° weakened after reduction by ascorbic acid, whereas a new peak appeared at 2θ = 25.4°, indicating the formation of RGO. These peaks confirm the successful reduction of GO to RGO using ascorbic acid [65]. The XRD patterns of pristine BC displayed main peaks at 2θ = 14.6°, 16.8°, and 22.8°, corresponding to the (110), (101), and (020) diffraction planes of the cellulose I structure, respectively [66]. Ppy showed a broad peak around 2θ = 25.55°, indicating an amorphous structure. In the BC/Ppy nanocomposite, the characteristic BC peaks disappeared, and a new peak at 2θ = 24.3° emerged, which was attributed to amorphous Ppy. With the addition of RGO, the BC/Ppy/RGO nanocomposite exhibited weaker peaks in the ternary composite, and the broad peak at 2θ = 24.3° suggested the presence of RGO. This indicates that the crystallization behavior of BC and RGO is hindered by amorphous Ppy.

Figure 3B,C show the FTIR spectra illustrating the impregnation of RGO into the BC matrix, polymerization of Ppy along the 3D web-like fibril architecture of BC, and immobilization of phage particles on the BC/Ppy/RGO biointerface. The characteristic peaks for -OH appeared at 3346 cm^−1^, while bands corresponding to the symmetric and asymmetric vibrations of C-H were observed at 2895 cm^−1^. The peak at 1643 cm^−1^ corresponds to the C=O stretching vibrations of the glucose carbonyl group. Peaks related to CH_2_ and CH_3_ deformation appeared between 1427 and 1470 cm^−1^, the peak for C-O-C deformation vibration appeared at 1161 cm^−1^, and the C-O stretching vibration appeared at 1060 cm^−1^ [67]. Several new peaks associated with Ppy were detected in the BC/Ppy composite. The peak at 1522 cm^−1^ is ascribed to the N-O stretching vibrations of a nitro compound, while the peaks at 1437 and 1278 cm^−1^ are attributed to C=C stretching and C-N stretching vibrations of the pyrole ring, respectively [68,69]. In the BC/Ppy/RGO composite, the characteristic peaks of the pyrrole ring remained consistent, but the main peaks, such as those for C=C, shifted to higher frequencies. This shift suggests an interaction between Ppy and RGO, likely due to π-π stacking interactions and hydrogen bonding [70,71]. The spectrum of the BC/Ppy/RGO–phage biointerface showed an increase in peak intensity and emergence of additional peaks after phage immobilization. The observed peaks include 1629 cm^−1^ for N-H bending, 1537 cm^−1^ and 1518 cm^−1^ for N-O stretching, 1473 cm^−1^ for C-H bending, 1275 cm^−1^, 1169 cm^−1^, and 1077 cm^−1^ C-C for C-C stretching, 1022 cm^−1^ for C-N stretching of amines, and 962 cm^−1^ for C=C bending. These characteristic FTIR peaks indicate a strong interaction between the BC/Ppy/RGO composite and phage proteins, as well as the involvement of various functional groups in phage immobilization [72]. Additionally, the amino acid sequences in the phage head proteins, such as aspartate and glutamate, which contain -COOH groups, interact with positively charged Ppy on the surface. This suggests possible electrostatic interactions between the phage head and Ppy, as well as amide bonding between -COOH groups in phage head proteins and amine groups on the biointerface [73,74,75].

XPS was employed to further analyze the elemental composition and chemical structure of the BC–phage-based biointerface (Figure 3D). Comperative wide-scan XPS spectra confirmed the presence of C, O, and N in the BC/Ppy/RGO composite, both with and without phages. Additionally, all the nanocomposites—BC, BC/Ppy, BC/Ppy/RGO, and the phage-immobilized BC/Ppy/RGO—exhibited binding energies corresponding to the carbon 1s (C 1s) and oxygen 1s (O 1s) core shells. Notably, there was a significant increase in the carbon content after the polymerization of Ppy and impregnation of RGO (Table S2). In the BC/Ppy/RGO–phage biointerface, the N 1s core peak was deconvoluted into three components at 401.6, 400.01, and 398.2 eV, corresponding to graphatic (30.8%), pyrrolic (68.7%), and pyridine nitrogen (0.49%), respectively (Figure 3E). The high content of pyrrolic nitrogen in the interfacial structure is particularly beneficial, as it enhances conductivity and promotes charge-directed phage immobilization, thereby increasing the conductivity of the electrode, whereas pyridine nitrogen contributes to efficient phage immobilization [76].

### 3.3. Anti-Salomenlla Activity of the Phage-Based Biointerface

**Figure 4:**
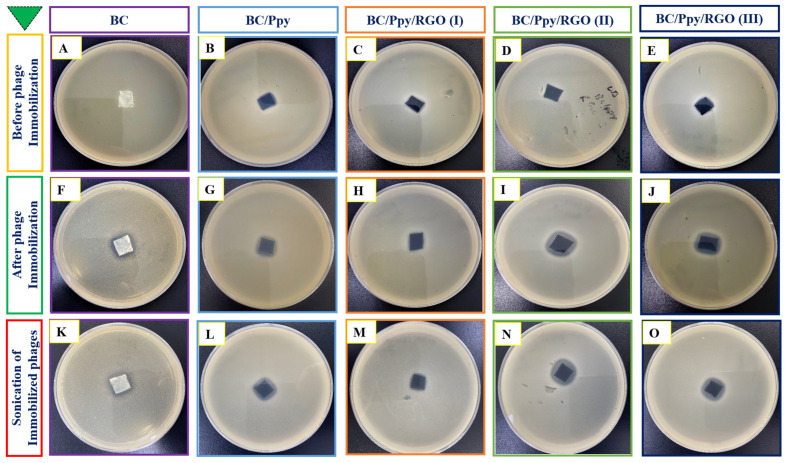
Plaque assays for anti-*Salmonella* potential of BC-based biointerfaces. (**A**–**E**) Plaque formation of materials such as BC, BC/Ppy, and BC/Ppy/RGO (I–III). (**F**–**J**) Plaque formation after immobilization of phages on different BC-based bio-interfaces such as BC, BC/Ppy, and BC/Ppy/RGO (I–III)–phage. (**K**–**O**) Sonicated interfaces with immobilized phages and their lytic activity.

The immobilization of phage particles on any surface does not automatically guarantee effective bacterial capture or antibacterial activity. For successful bacterial recognition and capture, the orientation of phage recognition proteins at the tail position is crucial [77]. Therefore, it was necessary to confirm the effectiveness of phage immobilization against *S. typhimurium* on the surface of the BC/Ppy/RGO biointerface both before and after external stimulation (sonication), following a standard protocol [78]. The BC film was modified with RGO and Ppy to make it more conductive. The positive charge of the polycationic Ppy on the surface enhances the attraction of the negatively charged phage heads on the capsid [79]. To promote charge interaction and enable physical adsorption of the phages onto the BC/Ppy/RGO surface, the strips were incubated in a phage suspension with a concentration of 10^9^ PFU/mL in 10 mL of PBS, with gentle shaking. After immobilization, the strips were subjected to the disk diffusion method to observe the infection dynamics, and the efficiency of phage immobilization on different BC-based biointerfaces was evaluated using plaque assays. The assay results showed no plaque formation for the non-immobilized BC, BC/Ppy, and BC/Ppy/RGO (I–III) biointerfaces, indicating that BC and its modified forms were non-toxic to *S. typhimurium* (Figure 4A–E). In contrast, films with immobilized phages, that is, BC/phage, BC/Ppy–phage, and BC/Ppy/RGO (I–III)–phage composites, showed clear plaque formation around the films. The BC–phage films exhibited minimal plaque formation owing to the limited physical adsorption of small phages. However, the BC/Ppy and BC/Ppy/RGO–phage (I–III) films displayed excellent plaque formation. Among these, the BC/Ppy/RGO–phage (II) film showed the largest clear zone and highest anti-*S. typhimurium* activity, suggesting that the presence of Ppy promoted phage immobilization (Figure 4F–J). To test the stability of the immobilized phages, phage-coated materials were sonicated for 20 min to remove weakly attached phages [78]. The plaque assay results showed that phages physically adsorbed onto BC were detached, resulting in no plaque formation, indicating that sonication effectively removed most weakly anchored phages. In contrast, the BC/Ppy–phage and BC/Ppy/RGO (I–III)–phage materials retained clear lytic zones (Figure 4K–O). These findings suggest that the strong electrostatic interaction between the positively charged Ppy and the negatively charged phage head proteins contributes to the stability of phages on the biointerface, making them less prone to detachment.

To further validate the recognition and capturing ability of *S. typhimurium*, immobilized phages on the BC-based modified films were immersed in 20 mL of *S. typhimurium* culture at a density of 10^8^ CFU/mL with shaking at 37 °C, and the OD_600_ was measured hourly. The results showed that the *S. typhimurium* in the control group (without phages) reached normal growth and became static after 9 h. In contrast, *S. typhimurium* exposed to phage-treated films exhibited complete lysis of bacterial cells within 3 h, with the BC/Ppy and BC/Ppy/RGO (II)–phage films showing the most significant effects. These findings suggest that the infection dynamics of Ppy-polymerized cationic films and free phage were nearly identical, indicating sufficient and correct immobilization, maximizing the recognition and capture of *S. typhimurium* cells. Additionally, the infection dynamics of BC/phage and BC/Ppy/RGO–phage (I and III) were comparable, but the BC/Ppy/RGO (II) film demonstrated superior performance against *S. typhimurium*. This suggests that the modification of BC with 0.1% RGO did not fully cover the surface, leading to partial entrapment of phages, whereas 1% RGO completely covered the porous structure. However, with 0.5% RGO, the BC membrane retained its porous morphology, allowing phages to remain unimpeded. This indicates that the phage tails were free to recognize and capture *S. typhimurium* cells effectively. In conclusion, BC composites modified with Ppy and RGO showed excellent anti-*Salmonella* and lytic performance in both plaque and infection dynamics assays. The BC/Ppy/RGO-II biointerface proved to be ideal for active phage immobilization, ensuring that the phage heads remained attached to the surface, whereas tail RBPs were free for bacterial cell capture. Even after 20 min of sonication, the immobilized phages on the surface retained strong attachment and lytic activity.

### 3.4. Phage Density on BC/Ppy/RGO Biointerface

**Figure 5:**
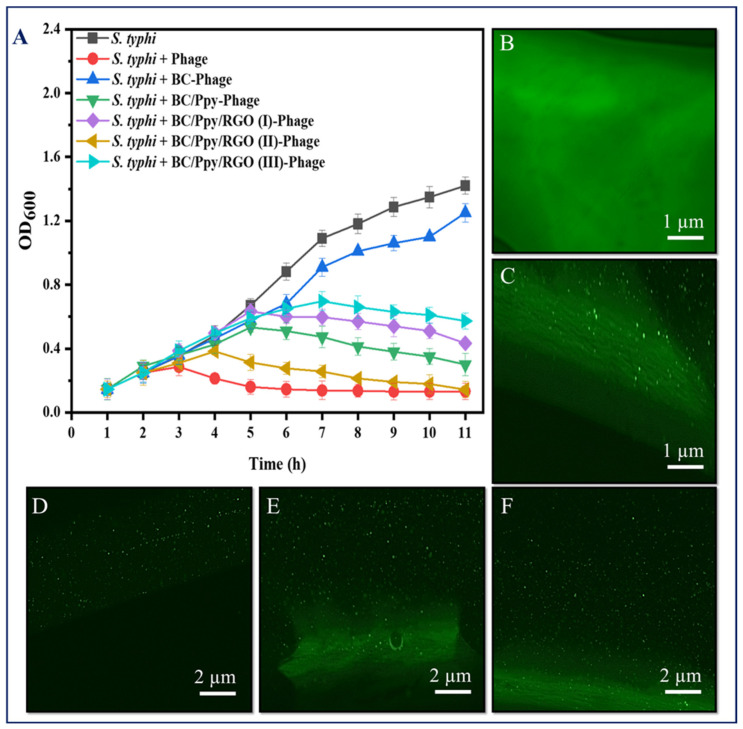
Infectious dynamics of phages immobilized on surface-modified BC against *S. typhimuruim* and their density under confocal microscopy. (**A**) Growth reduction curves in terms of optical density (OD_600_) of *S. typhi*, with free phages and phages immobilized on BC, BC/Ppy, BC/Ppy/RGO (I-III), (**B**) pristine BC, and (**C**) immobilized phages on BC (in-focus). (**D**–**F**) Density of stained phage particles (in-focus), while the composite is out of focus; (**D**) immobilized phages on pristine BC, (**E**) immobilized phages on BC/Ppy, and (**F**) immobilized phages on BC/Ppy/RGO.

Given the successful immobilization and impregnation of phages on the BC/Ppy/RGO surface, we further investigated the density and distribution of *S. typhimurium*-specific phages on BC-based modified films. Phage DNA was stained and analyzed using fluorescence microscopy because the antibacterial effectiveness of phages is closely related to their density and distribution on the surface (Figure 5B–F). High-density, well-oriented, and uniformly distributed phages are preferred because of their superior antibacterial activity [8,78,80]. Confocal microscopy revealed that the pristine BC had a fibrous structure (Figure 5C). When phages were attached to the BC, they were clearly visible in the focused images, confirming successful attachment to the cellulose fibers (Figure 5D). The phage densities on the BC/Ppy–phage and BC/Ppy/RGO–phage films were examined, where the phages were in focus while the films were unfocused. The results showed a higher density of phage particles on the films polymerized with polycationic Ppy. In the confocal microscopic images of the immobilized phages on the modified BC-based biointerface (Figure 5D), each fluorescence point represents a single phage particle. These results suggest that the positively charged BC-modified films promote active orientation of the phage heads towards the film surface, leading to densely populated phage attachments (Figure 5E,F). The fluorescent spots were counted using ImageJ software, showing that the density of phage particles/µm^−2^ on BC/phage, BC/Ppy–phage, and BC/Ppy/RGO–phage films was 2.1 ± 0.49, 5.2 ± 1.53, and 7.8 ± 1.81 particle/µm^−2^. The phage densities on the BC/Ppy/RGO–phage films were comparable to those of previously reported BC/PEI and polycaprolactone films [8,53]. The high density of phage particles is likely due to the fibrous, porous, and web-like architecture of the BC membrane combined with the polymerization of Ppy on its surface, which facilitates strong electrostatic interactions.

### 3.5. Electrochemical Characterization and Optimization of BC/Ppy/RGO Electrode

**Figure 6:**
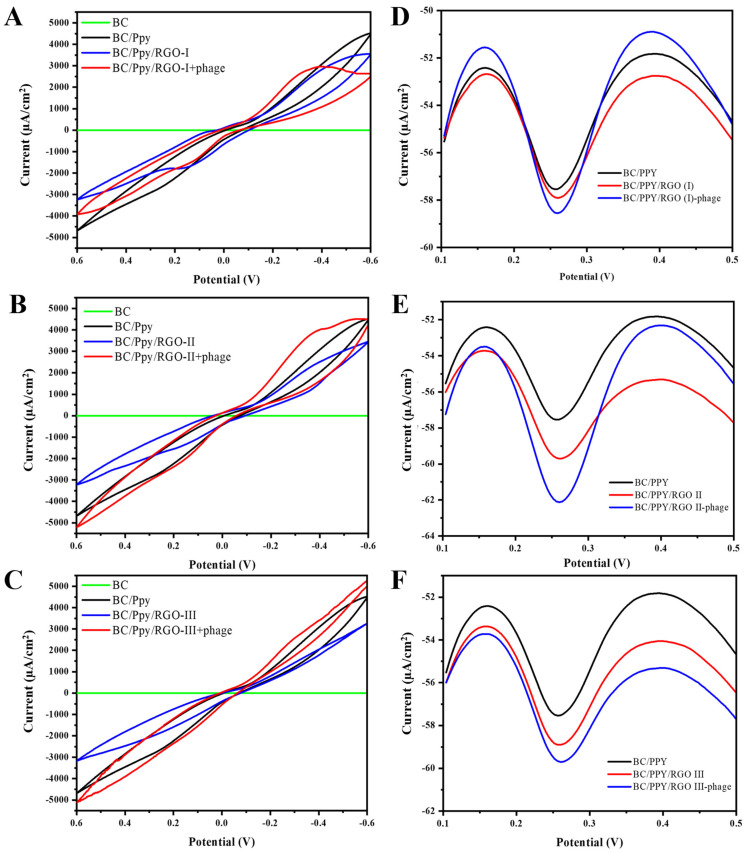
Electrochemical characterization of the surface BC-based modified electrodes for *S. typhi* detection. CV curves of different BC and BC-modified electrodes in cyclic voltammograms of the Fe(CN)_6_^3−^/Fe(CN)_6_^4−^ redox system, such as BC/Ppy, and different concentrations of RGO and immobilized phages, including (**A**) BC/Ppy/RGO (I)-phage, (**B**) BC/Ppy/RGO (II)-phage, and (**C**) BC/Ppy/RGO (III)-phage. DPV-based current responses of different concentrations of RGO with Ppy and phages (**D**) BC/Ppy/RGO (I)-phage, (**E**) BC/Ppy/RGO (II)-phage, and (**F**) BC/Ppy/RGO (III)-phage.

The electrochemical properties of the different BC-based electrodes were characterized using CV and DPV. The CV curves for pristine BC, BC/Ppy, and BC/Ppy/RGO with varying concentrations of RGO (I, II, and III), both with and without phages, are shown in Figure 6A–C.

The CV results demonstrate that the pristine BC had no current response, whereas BC/Ppy showed a significant improvement in conductivity (Figure 6A). The polymerization of Ppy and impregnation of RGO into the BC matrix greatly enhanced conductivity. These findings are consistent with those of previous studies, which showed that surface modification of BC through Ppy polymerization and RGO incorporation increased the current response [81,82]. Additionally, an increase in the current response was observed when phage particles were immobilized on the electrode surface [83,84], and the redox potential in the medium was reduced [85,86]. This evidence supports our understanding of electron/charge flows in the BC/Ppy/RGO–phage electrode. Further analysis showed that the BC/Ppy/RGO (I)–phage electrode had a lower current response than the II and III variants (Figure 6A), with the BC/Ppy/RGO (II)–phage exhibiting the highest current response, which further increased upon phage immobilization (Figure 6B). The current response of BC/Ppy/RGO (I) was higher than that of the BC/Ppy/RGO (I)–phage electrode but lower than that of the BC/Ppy/RGO (II)–phage electrode (Figure 6C).

The DPV results displayed similar characteristics to those of the CV results (Figure 6D–F). Notably, all the BC-based modified electrodes showed current responses, regardless of the RGO concentration. The DPV current for BC/Ppy was lower than that for the RGO variants. The BC/Ppy/RGO (I)-phage electrode maintained a consistent performance and was found to be the most efficient for DPV measurements (Figure 6E). Although the BC/Ppy/RGO (III)–phage electrode showed a comparable current response, it was less efficient than the BC/Ppy/RGO (II)–phage electrode (Figure 6D,F). Considering all the previous findings, such as phage density, CV, and DPV current responses, the BC/Ppy/RGO (II)–phage composite emerged as the most efficient and was therefore selected for the subsequent experimnets. Moreover, BC/Ppy/RGO (II)–phage showed a significantly higher current after phage particle immobilization than the other two composites.

### 3.6. Development of BC/Ppy/RGO–Phage Biosensor

#### 3.6.1. Optimization of Biosensor with Respect to Time and pH

To achieve optimal performance for *S. typhimurium* detection, key parameters, such as incubation time and pH, were tested and optimized using DPV.

The time required for phages to capture and recognize bacterial cells is directly influenced by these parameters. Therefore, the response time and sensitivity of the biosensor were assessed by incubating the BC/Ppy/RGO (II)–phage in PBS containing *S. typhimurium* at a concentration of 5.5 × 10^4^ CFU/mL. The DPV current response of the BC/Ppy/RGO-phage electrode was recorded in PBS, both in the absence (reference) and presence of *S. typhimurium*, and the responses to varying cell densities were compared (Figure S7A). The electrochemical signal in PBS alone was lower than that signal measured after adding the bacteria for 10 min. A slight increase in the current response was observed between 15 and 25 min. This increase is likely due to the deposition of *S. typhimurium* on the electrode surface, where immobilized phages are present. The attachment of bacteria can alter the local charge distribution on the sensor surface, affecting electron transfer kinetics and leading to an increased current. Additionally, the proximity of the captured bacteria to the electrode facilitates electron transfer between the bacterial cells and the electrode. Moreover, the attachment of pathogens to the phages may trigger enzymatic reactions that deliver genetic material and initiate other signaling mechanisms, further increasing the current output [86,87,88,89]. A more pronounced increase in the current response was observed at 30 min, which was likely due to the maximum number of bacterial cells captured by the phages on the electrode surface. A sudden and significant increase in the current response was detected at 35 min, continuing to 40 min, suggesting that the captured bacterial cells had been lysed, releasing cellular content and progeny phages. The presence of a higher concentration of cellular components and possibly progeny phages in the solution can enhance electron transfer, leading to a maximum electrochemical response, as indicated by the sharp increase in the current [90]. Although the current response continued to increase between 40 and 55 min, this increase was relatively small compared to the surge observed between 35 and 40 min. This suggests ongoing bacterial capture and electron transfer, with phages actively capturing bacteria and facilitating electron transfer during this period, resulting in a sustained but small increase in the current response. The current response level was between 45 and 55 min and then decreased at 60 min. This leveling off likely indicates a saturation point, where most of the immobilized phages had captured bacteria, leading to a plateau in electron activity. The subsequent decrease in the current response at 60 min could be attributed to the complete lysis of all captured cells, with the release of all cellular contents and progeny phages reducing the overall electron transfer efficiency.

Phages are effective over a wide pH range (4–10) for infecting their host bacterial cells, making it likely that phage-based biosensors are pH-dependent for bacterial detection [91,92]. To evaluate this, the BC/Ppy/RGO–phage biosensor was used for the DPV-based detection of *S. typhimurium* at different pH values (4–10), with an optimal response time of 30 min, and the current responses were recorded. The DPV results showed that the lowest current responses occurred at the extreme pH values of 4 and 10. This could be due to unfavorable conditions for either the phage-mediated capture of *S. typhimurium* or the electrochemical process. The highest current response was observed at pH 7, followed by pH 6 and pH 8. These findings indicate that a neutral pH provides optimal conditions for both phage-mediated bacterial capture and subsequent electrochemical detection. Additionally, the results demonstrated that the current responses gradually decreased as the pH moved away from neutral, either lower or higher. This suggests that the performance of the biosensor was influenced by the pH of the surrounding medium, as illustrated in Figure S7B. Overall, the DPV results confirmed that, while the biosensor was functional across a broad pH range, the optimal current response was achieved at pH 7.0.

**Figure 7:**
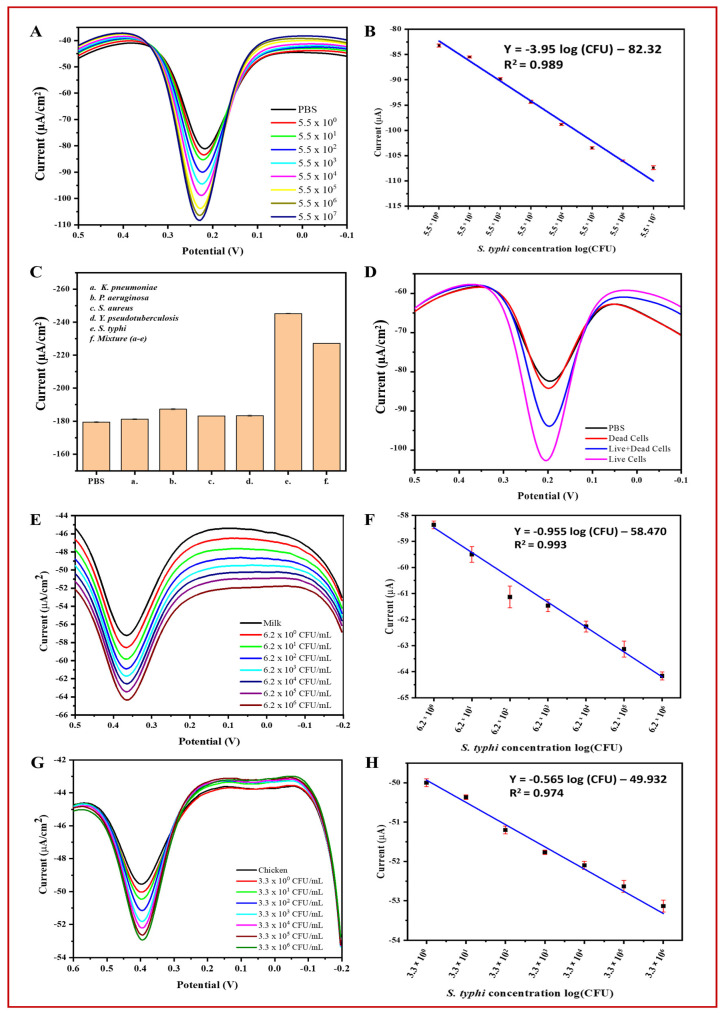
Specificity, live/dead cell discrimination, and practical applications of the BC/Ppy/RGO-phage biosensor for electrochemical detection of *S. typhi*. (**A**) DPV current response towards detected *S. typhi* in PBS, (**B**) linear range of *S. typhi* detection in PBS, (**C**) specificity of the biosensor, (**D**) biosensor discrimination for live, dead, and mixture of live/dead *S. typhi*, (**E**) DPV curves of the detected *S. typhi* in milk, (**F**) linear range of *S. typhi* in milk, (**G**) detection of *S. typhi* in chicken samples at different concentrations, and (**H**) linear range of detection in chicken. The standard deviations of triplicate analyses for each experiment are indicated by error bars.

Using the optimized conditions, *S. typhimurium* was detected in PBS using the BC/Ppy/RGO-phage biosensor across a concentration range of 5.5 × 10^0^ to 5.5 × 10^7^ CFU/mL using DPV (Figure 7A). The DPV curves showed that the peak current increased with increasing concentrations of *S. typhimurium*. This increase in the current response is likely due to the capture of bacterial cells by phages, leading to cell lysis and the subsequent release of cellular contents and progeny phages [93,94]. The release of these cellular components and progeny phages into the solution enhances electron transfer, resulting in a significant increase in electrochemical response [17,90]. Progeny phages further contribute to current enhancement by capturing additional free bacterial cells and generating a stronger signal. Additionally, the limit of detection (LOD) was calculated based on the linear correlation over a concentration range of 10^0^–10^7^ CFU/mL (Figure 7B). The bacterial density was determined using the following linear regression equation: Y = −3.95 log (CFU) − 82.32 (R^2^ = 0.989). The LOD for *S. typhimurium* was calculated using Equation (1) [17]:

LOD = 3 σ/m(1)

where “σ” and “m” represent the standard deviation of the blank (PBS) and slope of the calibration curve, respectively. The standard deviation of the blank was 0.971, the slope was 3.95, and the LOD of *S. typhimurium* was determined to be 1 CFU/mL with a linear detection range of 10^0^–10^7^ CFU/mL in 10 mL PBS.

#### 3.6.2. Specificity, Reproducibility, and Stability of the BC/Ppy/RGO–Phage Biosensor

To assess the selectivity of the biosensor, we measured the DPV-based current response of the developed phage-based biosensor for *S. typhimurium* in the presence of non-host bacterial strains and in a mixture containing all these strains. The results demonstrated that the highest current response was observed for the host bacterial *S. typhimurium*, followed by the mixture of all the bacterial strains. In contrast, the response to non-specific bacteria was negligible compared to that of the control (PBS). Figure 7C illustrates the current response curves for PBS, host bacterial strains, and non-host bacterial strains.

Reproducibility is crucial for practical application and on-site detection of target bacterial pathogens. We evaluated the reproducibility by measuring the ΔI values across five different electrodes, each exposed to the same concentration of *S. typhimurium* (10^6^ CFU/mL). The DPV current responses from these tests showed consistent relative responses, confirming acceptable reproducibility (Figure S8A).

The BC/Ppy/RGO–phage biosensors were stored at 4 °C, and their DPV-based current response was measured weekly for five weeks to assess their stability. The biosensor demonstrated excellent stability, with consistent current responses observed throughout the entire period (Figure S8B). These results suggest that the biosensor is well suited for extended use, making it a promising option for on-site detection and other applications. The durability is likely attributed to the inherent stability of the BC hydrogel and its humid environment, which helped preserved the viability of the phage components.

#### 3.6.3. Biosensor Application in Real Samples and Discrimination of Live/Dead Cells

Detecting target analytes in real samples is crucial for assessing biosensor efficiency. Therefore, we evaluated the feasibility and practical applications of the developed phage-based biosensor in real samples of *S. typhimurium* in milk and chicken. The DPV-based results showed that the peak current increased with increasing concentrations of bacterial cells (ranging from 10^0^ to 10^6^ CFU/mL). This indicates that the biosensor is effective and feasible for the analysis of real samples. However, the current response in real samples (i.e., milk and chicken) was lower than that in PBS, likely because of the complex nature of these samples (Figure 7E–F). The biosensor was able to detect *S. typhimurium* at concentrations up to ~10^7^ CFU/mL in PBS and ~10^6^ CFU/mL in milk and chicken. Similar to PBS, a linear correlation and current response were observed in milk and chicken (Figure 7G,H) within the range of 10^0^–10^6^ CFU/mL. The density of *S. typhimurium* in milk and chicken was calculated using the following linear regression equations: milk: I (μA) = −0.955 log (CFU) − 58.470 (R^2^ = 0.993) and chicken: I (μA) = −0.565 log (CFU) − 49.932 (R^2^ = 0.974). The LOD for *S. typhimurium* was determined using the slope of the calibration curve and Equation (1) [17].

In Equation (1), “σ” and “m” represent the standard deviation of the milk and chicken and the slope of the calibration curve, respectively. The standard deviation of milk was 1.33, with a slope of −0.955, and for chicken, it was 0.45 with a slope of −0.565. The LOD of *S. typhimurium* was determined to be 5 CFU/mL in milk and 3 CFU/mL in chicken within the linear range of detection of 10^0^–10^6^ CFU/mL in 10 mL samples.

To further validate the accuracy of the phage-based biosensor, *S. typhimurium* cells were inoculated into PBS, milk, and chicken samples and analyzed using both the developed biosensor and plate count method. Accuracy was assessed by comparing the recovery percentages (Table S3). The results showed that the biosensor was comparable to the plate count method, demonstrating high accuracy and meeting the key requirements for detecting *S. typhimurium* in real samples. *The biosensor produced* comparable results (Figure S9). Additionally, to evaluate the ability of the biosensor to discriminate between live and dead bacterial cells as well as a mixture of live/dead cells, the DPV-based current response was measured. The results showed that PBS and dead cells produced similar current peaks, whereas live cells and a mixture of live and dead cells generated a significantly higher current response (Figure 7D). These findings confirm their efficiency and reliability.

Overall, the developed phage-based biosensor demonstrated excellent sensitivity, accuracy, selectivity, and the ability to distinguish between live and dead bacterial cells. A summary of its performance, including efficiency, range, accuracy, and LOD, compared with recent reports on *S. typhimurium* detection, is provided in Table 1.

## 4. Conclusions

This study successfully developed an ultrasensitive electrochemical biosensor for the detection of *Salmonella typhimurium* in various food matrices by utilizing surface-modified bacterial cellulose (BC) integrated with polypyrrole (Ppy), reduced graphene oxide (RGO), and immobilized phage particles. The innovative design of the BC/Ppy/RGO–phage composite material resulted in a highly conductive, flexible, and efficient biointerface for bacterial detection. The key conclusions drawn from the study include the following:

High sensitivity and low detection limits: The biosensor demonstrated an exceptionally low detection limit of 1 CFU/mL in PBS and maintained low detection limits in real food samples, achieving 5 CFU/mL in milk and 3 CFU/mL in chicken. This sensitivity underscores the ability of the biosensor for early and accurate detection of *S. typhimurium*, crucial for ensuring food safety.

Broad detection range: The sensor exhibited a wide linear detection range from 10^0^ to 10^7^ CFU/mL, allowing for the detection of varying concentrations of *S. typhimurium* in different food samples. This range enhances the applicability of the biosensor in diverse food safety scenarios.

Selectivity and discrimination: The biosensor effectively discriminated between live and dead bacterial cells, which is an essential feature for practical food safety monitoring. The specificity conferred by the immobilized phage particles contributed significantly to this selectivity.

Stability and versatility: The biosensor maintained its performance over a broad pH range (4–10) and remained stable for up to six weeks, highlighting its robustness and potential for long-term application in various food safety contexts.

Practical application: The successful application of the biosensor in real food matrices (milk and chicken) demonstrated its practical viability and potential for integration into food safety monitoring systems.

Overall, the BC/Ppy/RGO–phage biosensor is a promising tool for the rapid, sensitive, and selective detection of *S. typhimurium* in food matrices, offering significant advancement in food safety technologies. Further development and optimization could pave the way for its deployment in commercial food safety monitoring and quality control.

## Figures and Tables

**Table 1:** The modified BC/Ppy/RGO–phage-based biosensor showed excellent performance compared with recently reported biosensors for detecting *S. typhi*.

Substrate	Detection Method	Recognition Element	Linear Range (CFU·mL^−1^)	Detection Time (min)	Limit of Detection (CFU·mL^−1^)	Sample Type	Live/Dead Cell Discrimination	Ref.
NPG/Au/GCE	EIS	Aptamer	6.5 × 10^2^–6.5 × 10^8^	40	1	Egg	No	[95]
Pty-Au NPs	Capacitive	Phage	2.0 × 10^2^–1.0 × 10^7^	40	200	Chicken	No	[96]
GNP/GO	Electrochemical	Phage RBP 41 IS A	3–3 × 10^6^	30	2	PBS, Milk	No	[97]
CoFe-MOFs-graphene	Electrochemical	Antibody	2.4 × 10^2^–2.4 × 10^8^	90	1.2 × 10^2^	Milk	No	[98]
Det7/Au	SPR	Det7 phage tail protein	0.5 × 10^4^–5.0 × 10^7^	20	-	Apple juice	No	[99]
E-CRISPR	PCR	DNA	6.7 × 10^1^–6.7 × 10^5^	360	55	Poultry	No	[100]
BC/Ppy/RGO	Electrochemical	Phage	5.5 × 10^0^–5.5 × 10^7^	30	1	PBS	Yes	Current study
			6.2 × 10^0^–6.2 × 10^6^		5	Milk		
			3.3 × 10^0^–3.3 × 10^6^		3	Chicken		

Au = gold; Pty = polytyramine; NPG = nonporous gold; SPR = surface plasmon resonance; Det7 = Det7 phage tail proteins; PCR = polymerase chain reaction; CRISPR = clustered regularly interspaced short palindromic repeats; MOFs = metal–organic frameworks; RGO = reduced graphene oxide; BC = bacterial cellulose; Ppy = polypyrrole.

## Data Availability

The original contributions presented in the study are included in the article/Supplementary Materials, further inquiries can be directed to the corresponding authors.

## Supplementary Materials

The following supporting information can be downloaded at https://www.mdpi.com/article/10.3390/bios14100500/s1: Figure S1: Schematic illustration of the stepwise modification of the pristine BC and fabrication of the BC/Ppy/RGO. The pure BC (white), modified BC (black), and Immobilization of bacteriophages on biointerface for *S. typhi* detection using the electrochemical workstation; Figure S2: Schematic illustration of the isolation, purification and specificity of the *S. typhi* phages; Figure S3: *S. typhi* phage spot and plaque formation; Figure S4: TEM micrograph of the *S. typhimurium* phage at (A) 500 nm and (B) 200 nm resolution; Figure S5: FE-SEM micrograph of the Ppy polymer after chemical in-situ oxidative polymerization; Figure S6: BC and modified BC/Ppy/RGO composite, (A) Pristine-BC, and (B) Conductive interface, Bendable composite, and lightweight composite on a dog tail plant; Figure S7: BC/Ppy/RGO-phage biointerface optimization for *S. typhi* detection. (A) The response time and net current increase, and (B) the pH effects on the current responses; Figure S8: (A) Stability of the developed biosensor and its potential for *S. typhi* detection, and (B) Reproducibility of the BC/Ppy/RGO-phage biointerface; Figure S9: (A) Recovery of *S. typhi* by plate count method, (A) single CFU at the corner edge recovered from PBS, (B) 3 CFU from Milk, and (C) 3 CFU from beef in 48–72 h, prospectively; Table S1: The isolated phage lytic activity against different bacterial strains; Table S2: XPS elemental analysis with detailed information; Table S3: Recovery tests for *S. typhi* in PBS, milk, and chicken samples by BC/RGO/Ppy-phage based biosensor and plate count.
