# Supplementary material for: Ultrasensitive Electrochemical Detection of Salmonella typhimurium in Food Matrices Using Surface-Modified Bacterial Cellulose with Immobilized Phage Particles
Source: Biosensors (Basel). 2024 Oct 14;14(10):500. doi: 10.3390/bios14100500 (PMC11506579; doi:10.3390/bios14100500)
Supplement: Supplementary file 1 [file biosensors-14-00500-s001.zip › biosensors-3210916-supplementary.pdf]

Supporting Information

Ultrasensitive Electrochemical Detection of *Salmonella typhimurium* in Food Matrices Using Surface-Modified Bacterial Cellulose with Immobilized Phage Particles

Wajid Hussain [1,†], Huan Wang [1,†], Xiaohan Yang [1], Muhammad Wajid Ullah [2,*], Jawad Hussain [3], Najeeb Ullah [4], Mazhar Ul-Islam [5], Mohamed F. Awad [6] and Shenqi Wang [1,*]

[1] Advanced Biomaterials and Tissue Engineering Center, College of Life Science and Technology, Huazhong University of Science and Technology, Wuhan 430074, China; wajidusafzai@hust.edu.cn (W.H.); m202071916@hust.edu.cn (H.W.); d202180745@hust.edu.cn (X.Y.)
[2] Department of Pulp & Paper Engineering, College of Light Industry and Food Engineering, Nanjing Forestry University, Nanjing 210037, China
[3] Department of Biomedical Engineering, College of Life Science and Technology, Huazhong University of Science and Technology, Wuhan 430074, China; jawadusafzai5@gmail.com
[4] Department of Chemical and Biomolecular Engineering, University of Tennessee, Knoxville, TN 37996, USA; lzn122@utk.edu
[5] Department of Chemical Engineering, Dhofar University, Salalah 211, Oman; mulislam@du.edu.om
[6] Department of Biology, College of Science, Taif University, Taif 21944, Saudi Arabia; m.fadl@tu.edu.sa
* Correspondence: wajid_kundi@hust.edu.cn or wajid_kundi@njfu.edu.cn (M.W.U.); shenqiwang131@hust.edu.cn (S.W.)
† These authors contributed equally to this work.

1. Isolation, purification, and amplification of S. *typhi* specific-phage

The *S. typhimurium* lytic phage was isolated from sewage water pig form following a previously reported protocol [46] with slight modifications. Briefly, a 400 ml sewage water sample was collected from a dairy farm (Henan Province, China) and processed under static conditions to settle dense particles. The mixture was centrifuged at 5000 ^{x} g for 10 min at room temperature to remove solid debris and particles. The supernatant was filtered by a 0.22 μm syringe-driven filter and stored at 4°C for further use.

The filtrate was processed by inoculating the *S. typhimurium* cell suspension at the exponential phase in LB broth (approximately 10^7 CFU/mL) and was incubated at 37 °C for 22 ± 2 h to amplify potential phages. Briefly, *S. typhimurium* and filtrate (1:1) were

mixed and added to 5 mL of top agar tubes, poured onto bottom agar plates, and incubated at 37 °C for 12-16 h, and bacterial cell lysis was observed. Next, 10 mL of SM buffer was added, and the upper layer of soft agar was disrupted with a sterilized pipette tip, transferred to falcon tubes, and centrifuged at 5000 × g for 10 min at 4 °C to pellet the remaining agar and host bacteria. The supernatants were isolated and filtered, and the titer of the phage stock was measured by plaque assays using serial dilution (10^{-5}-10^{-9} PFU/mL) in PBS. The titer of phages was calculated using DLA, and the plates were labeled with dilution factors. The next day, phage plaques were counted, and the phage titer was calculated. The phages were stocked in PBS buffer at 4 °C for a short time like a month and in 50 % glycerol solution at -80°C for long-term preservation. Transmission electron microscopy (TEM; Hitachi, Japan) was used to study the morphology of isolated phages. Briefly, phage lysate (~10 µL, ~10^9 PFU/mL) was spotted on carbon grids for 10 min, negatively stained with 2 % phosphotungstic acid (PTA), and vacuum-dried. The grid was observed through TEM at 15 kV (500 nm resolution) and 80 kV (100 nm resolution).

During enrichment, the LB broth with phages and bacteria clearly showed lysis by phages. The phage lysate spotted on the bacterial lawn showed clear spots, which confirmed the presence of phages. Additionally, the filtrate dilution of 10^{-7} showed clear plaques (Fig. S2) and lytic activity was retained after storage at 4°C and − 80°C. The isolated phage showed properties similar to those of the family *Siphoviridae*, with an icosahedral head and long contractile tail. The head of the phage had a diameter of 86 nm and a tail length of 112 nm (Fig. S3).

To determine phage infectivity other than that of *S. typhimurium*, a phage spot assay was used to check the viability of the bacterial strains. Briefly, 100 µL of fresh bacterial culture of *S. typhimurium*, *S. aureus*, *Pseudomonus aerginusa*, *Yersinia pseudotuberculosis*, and *Klebsiella pneumonia* were mixed in 5 mL of upper liquid medium,

poured on an LB agar plate, and incubated for 15 min to solidify the medium. After lysis, 5 µL of the phage lysate was spotted onto the center of an LB plate and incubated at 37 °C overnight. Observation of the *S. typhi* host bacteria showed clear zones, and non-host strains such as *S. aureus*, *Pseudomonus aerginusa*, *Yersinia pseudotuberculosis*, and *Klebsiella pneumonia* did not show any clear zones, indicating that *S. typhimurium* is the host bacterium of the isolated phage (Table S1).

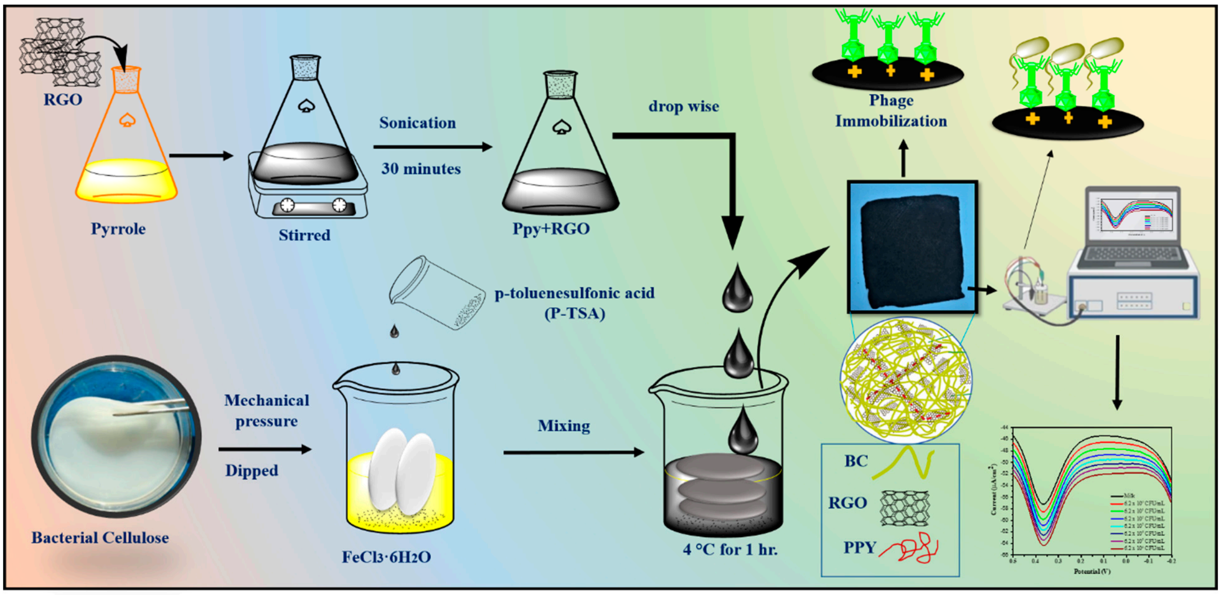

Figure S1: Schematic illustration of the stepwise modification of the pristine BC and fabrication of the BC/Ppy/RGO. The pure BC (white), modified BC (black), and Immobilization of bacteriophages on biointerface for *S. typhi* detection using the electrochemical workstation.

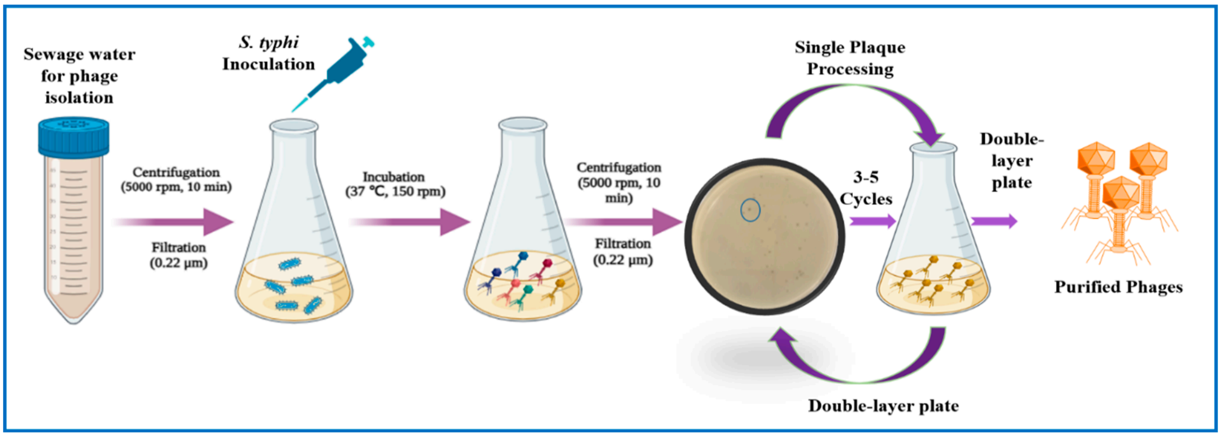

Figure S2: Schematic illustration of the isolation, purification and specificity of the *S. typhi* phages.

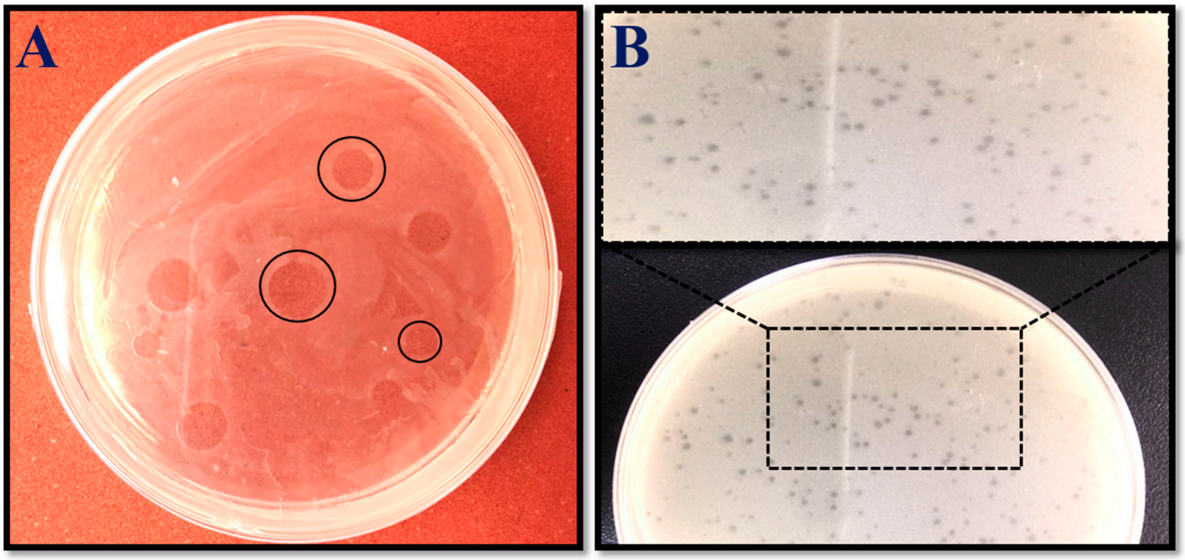

Figure S3. *S. typhi* phage (A) clear zone formation through spot assay, and (B) phage plaque formation.

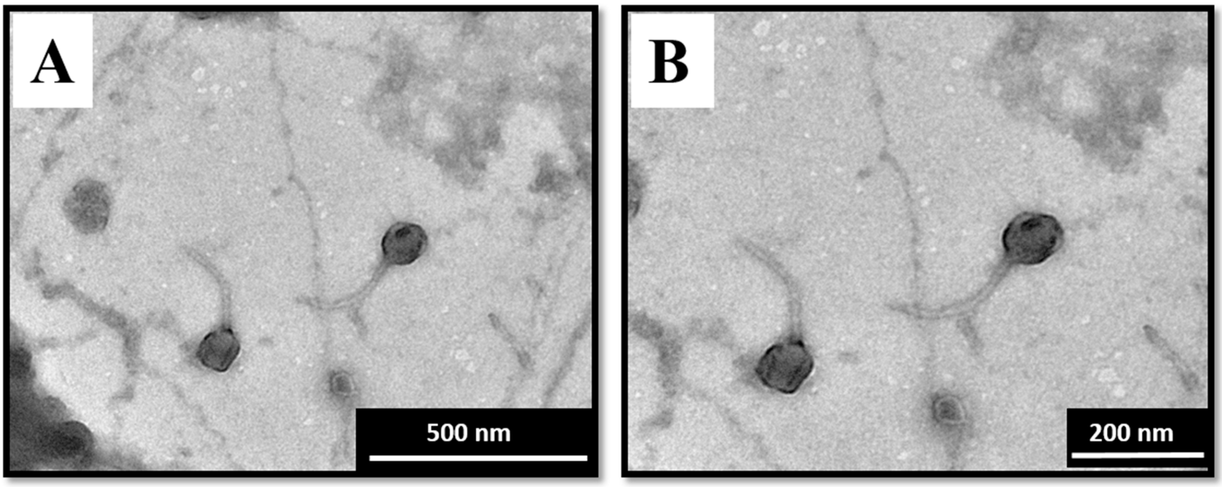

Figure S4. TEM micrograph of the *S. typhimurium* phage at A) 500 nm and B) 200 nm resolution.

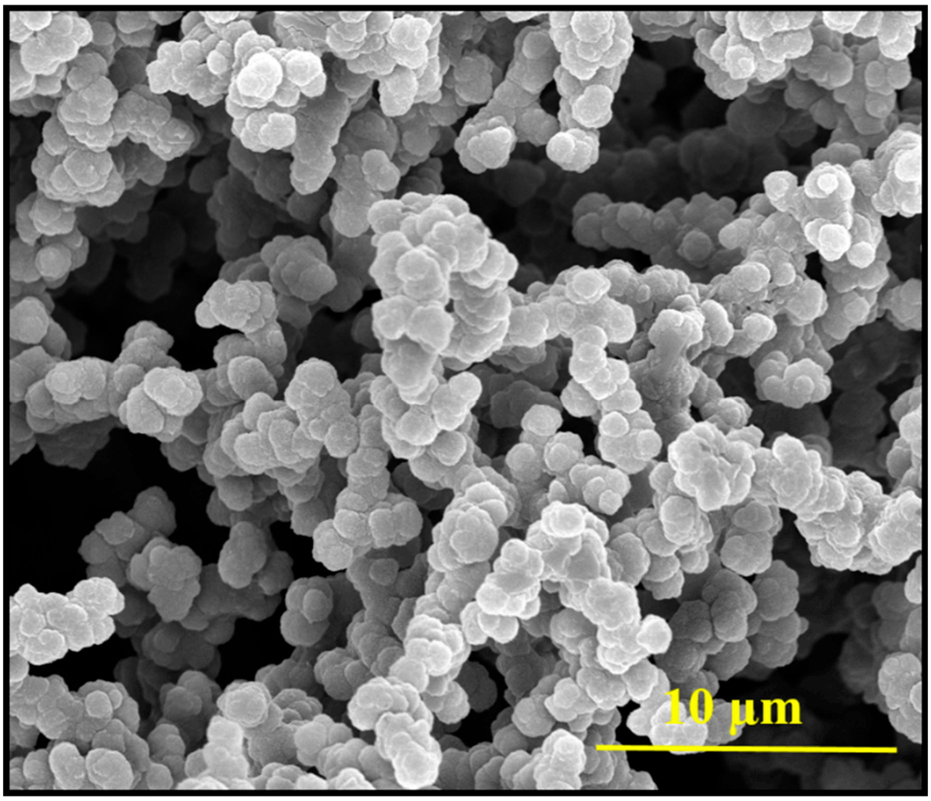

Figure S5. FE-SEM micrograph of the Ppy polymer after chemical in-situ oxidative polymerization.

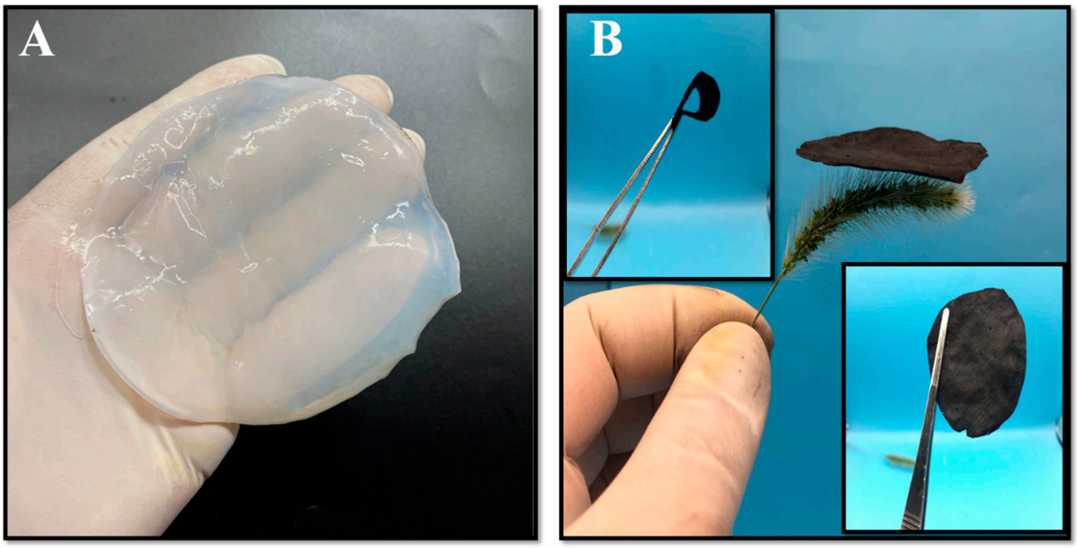

Figure S6. BC and modified BC/Ppy/RGO composite, **A)** Pristine-BC, and **B)** Conductive interface, Bendable composite, and lightweight composite on a dog tail plant.

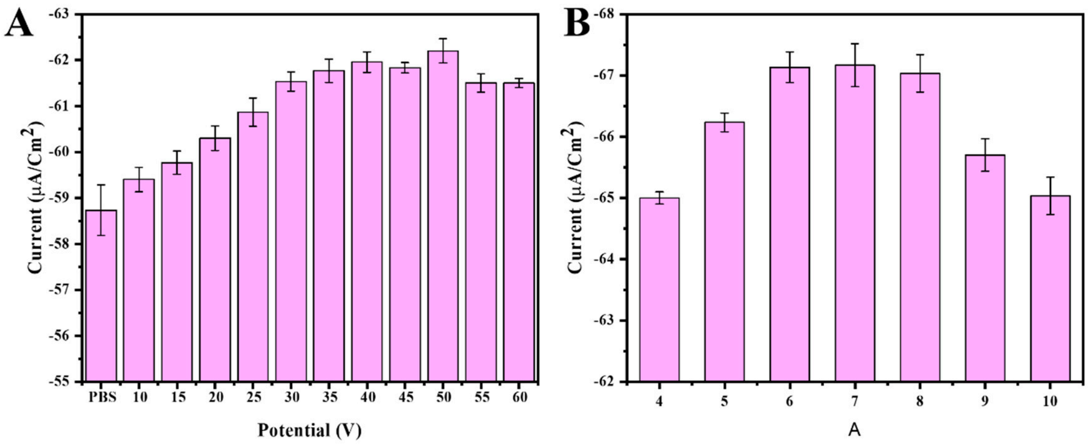

Figure S7. BC/Ppy/RGO-phage biointerface optimization for *S. typhi* detection. A) The response time and net current increase, and B) the pH effects on the current responses.

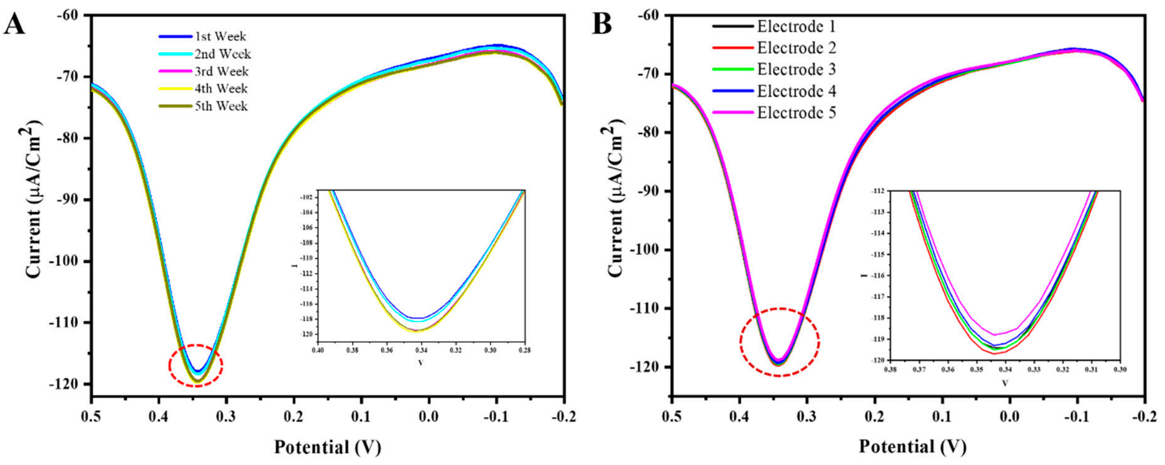

Figure S8. A) Stability of the developed biosensor and its potential for *S. typhi* detection, and B) Reproducibility of the BC/Ppy/RGO-phage biointerface.

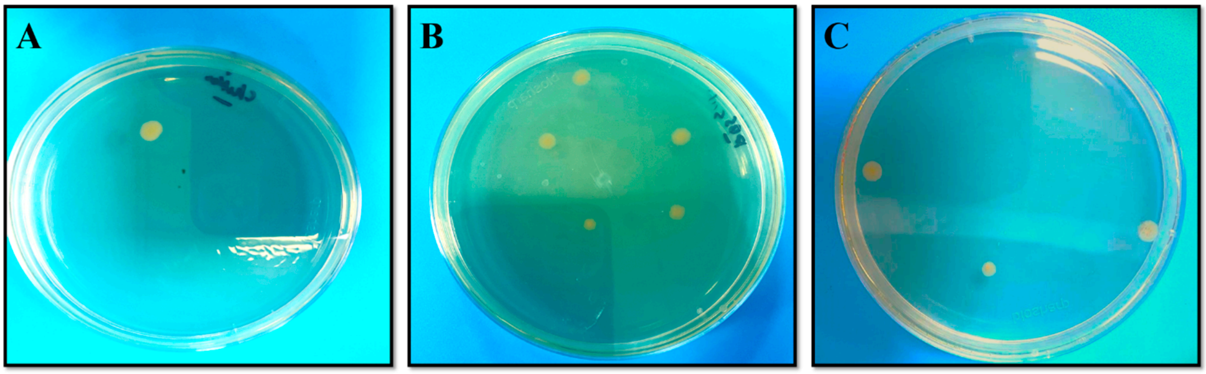

Figure S9. A) Recovery of *S. typhi* by plate count method, A) single CFU at the corner edge recovered from PBS, B) 3 CFU from Milk, and C) 3 CFU from beef in 48-72 hr., prospectively.

Table S1. The isolated phage lytic activity against different bacterial strains

Bacterial strain	Appearances of Plaques by DLA	Clear Zone

S. aureus (1.76 x 10^6 CFU/mL)	No	No
P. aeruginosa (3.07 x 10^6 CFU/mL)	No	No
K. pneumonia (2.05 x 10^6 CFU/mL)	No	No
Y. psedudotuberculosis (3.2 x 10^6 CFU/mL)	No	No
S. typhimuruim (4.2 x 10^6 CFU/mL)	**Yes**	**Yes**

Table S2. XPS elemental analysis with detailed information

Items	Peak	Position BE (eV)	Atomic Content (%)	Mass Content (%)
BC	O 1s	531.28	39.8	46.8
	C 1s	285.03	59.3	52.3
	N 1s	398.33	0.9	0.38
BC/Ppy	O 1s	533. 16	24.1	26.4
	C 1s	285.16	65.4	53.8
	N 1s	400.06	5.9	5.7
BC/Ppy/RGO	O 1s	531.82	17.4	19.0
	C 1s	285.17	68.4	56.0
	N 1s	400.12	8.6	8.2
BC/Ppy/RGO-phage	O 1s	531.56	31.1	31.3
	C 1s	285.06	53.6	40.4
	N 1s	399.01	9.0	8.0

Table S3. Recovery tests for *S. typhi* in PBS, milk, and chicken samples by BC/RGO/Ppy-phage based biosensor and plate count

Sample	Spiked (CFU.ml^{-1})	Detected (CFU.ml^{-1})		Recovery (%)[a]	
		Biosensor	Plate count	Biosensor	Plate count
PBS	5.5×10^3	5.43×10^3	5.54×10^3	98.7	100.7
Milk	6.2×10^3	$6. 17 \times 10^3$	6.23×10^3	99.5	100.4
Beef	3.3×10^3	3.23×10^3	3.4×10^3	97.7	100.3

[a] Recovery (%) is expressed as the ratio of the number of detected/number of spiked.

References

46. Jiang, L.; Zheng, R.; Sun, Q.; Li, C. Isolation, Characterization, and Application of Salmonella Paratyphi Phage KM16 against Salmonella Paratyphi Biofilm. *Biofouling* **2021**, *37*, 276–288, doi:10.1080/08927014.2021.1900130.
